# Investigations of socioeconomic factors associated with follow-up compliance with malaria treatment in Haiti

**DOI:** 10.26633/RPSP.2021.150

**Published:** 2021-12-10

**Authors:** Tamar E. Carter, Alexandre Existe, Madsen Beau de Rochars, Bernard A. Okech

**Affiliations:** 1 Baylor University Waco Texas United States of America Baylor University, Waco, Texas, United States of America; 2 Ministère de la Santé Publique et de la Population Port-au-Prince Haiti Ministère de la Santé Publique et de la Population, Port-au-Prince, Haiti; 3 University of Florida Gainesville Florida United States of America University of Florida, Gainesville, Florida, United States of America; 4 Uniformed Services University of the Health Sciences Bethesda Maryland United States of America Uniformed Services University of the Health Sciences, Bethesda, Maryland, United States of America

**Keywords:** Malaria, falciparum, clinical protocols, patient compliance, therapeutics, Haiti, Malaria falciparum, protocolos clínicos, cooperación del paciente, terapéutica, Haití, Malária falciparum, protocolos clínicos, cooperação do paciente, terapêutica, Haiti

## Abstract

**Objective.:**

To identify factors affecting compliance with follow-up during treatment in confirmed malaria patients at two health centers in Haiti.

**Methods.:**

A prospective observational study of malaria patients undergoing treatment over a six-week period. Patients’ return visits (follow-up visits) to the health centers for consultation in accordance with the physicians’ requests were recorded and used to determine compliance. Socioeconomic data were obtained from patient enrollment questionnaires and through post-treatment interviews. The management practices and procedures at the health centers to retain patients were also reviewed. Descriptive statistics and Spearman’s rank correlation were used to identify significant factors, which were used as variables in a logistic regression model.

**Results.:**

Sixty-eight percent of the malaria patients completed follow-up, with higher compliance being recorded in the larger, more established health center of Leogane (67%) than Cite Soleil (33%). The patient socioeconomic profiles differed between the two health center locations by level of education, religious diversity, household size, and percentage of married individuals. Crude logistic regression analyses identified health center location (OR = 0.179 [95% CI 0.064, 0.504]) and household size (OR = 1.374 [95% CI 1.056, 1.787]) to be associated with compliance. The adjusted model only identified health center location (OR = 0.226 [95% CI 0.056, 0.918]) as significantly associated with compliance.

**Conclusion.:**

Although patients’ household size may be important according to the crude logistic regression analysis, in the adjusted analysis the site location of the health center where patients receive treatment was identified as the only important factor associated with follow-up compliance in malaria patients during treatment in Haiti. This information might be helpful to improve treatment outcomes and contribute to the monitoring of antimalarial resistance in Haiti.

## RESUMEN

**Objetivo.:**

Determinar los factores que afectan el cumplimiento del seguimiento durante el tratamiento de los pacientes con malaria confirmada en dos centros de salud de Haití.

**Métodos.:**

Se llevó a cabo un estudio observacional prospectivo de los pacientes con malaria que recibían tratamiento a lo largo de un período de seis semanas. Se registraron las consultas subsiguientes de los pacientes a los centros de salud (consultas de seguimiento) de acuerdo con la solicitud de los médicos, que se emplearon para determinar el cumplimiento. Se obtuvieron los datos socioeconómicos de los cuestionarios del registro de pacientes y mediante entrevistas postratamiento. También se examinaron las prácticas y los procedimientos de gestión del centro de salud para promover la retención de los pacientes. Se emplearon estadísticas descriptivas y la correlación de rangos de Spearman para determinar los factores significativos, que se usaron como variables en un modelo de regresión logística.

**Resultados.:**

El 68% de los enfermos de malaria completaron el seguimiento. El mayor cumplimiento se registró en el centro de salud más grande y de mayor prestigio de Léogâne (67%) en comparación con el centro de Cité Soleil (33%). Los perfiles socioeconómicos de los pacientes difirieron entre las dos ubicaciones del centro de salud en lo concerniente al nivel de escolaridad, diversidad religiosa, tamaño del hogar y porcentaje de personas casadas. Los análisis crudos de regresión logística determinaron que había una asociación entre la ubicación del centro de salud (OR = 0,179 [IC de 95 % 0,064, 0,504]) y el tamaño del hogar (OR = 1,374 [IC de 95 % 1,056, 1,787]) con el cumplimiento. En el modelo ajustado se encontró que solo la ubicación del centro de salud (OR = 0,226 [IC de 95 % 0,056, 0,918]) estaba significativamente relacionada con el cumplimiento.

**Conclusión.:**

Aunque el tamaño del hogar de los pacientes podría ser importante según el análisis crudo de regresión logística, en el análisis ajustado se determinó que la ubicación del centro de salud en que los pacientes reciben el tratamiento era el único factor importante asociado con el cumplimiento de seguimiento de los pacientes con malaria en tratamiento en Haití. Es posible que esta información sea útil para mejorar los resultados del tratamiento y contribuir al seguimiento de la resistencia a los antimaláricos en Haití.

## RESUMO

**Objetivo.:**

Identificar os fatores que afetam a adesão ao seguimento durante o tratamento da malária em pacientes com diagnóstico confirmado em dois centros de saúde no Haiti.

**Métodos.:**

Estudo observacional prospectivo de pacientes com malária em tratamento durante um período de seis semanas. Os retornos dos pacientes (consultas de seguimento) aos centros de saúde para consulta, conforme solicitado pelos médicos, foram registrados e usados para determinar a adesão. Os dados socioeconômicos foram obtidos a partir dos cadastros dos pacientes e por meio de entrevistas pós-tratamento. As práticas e procedimentos de gestão para reter pacientes no centro de saúde também foram analisados. Foram utilizadas estatísticas descritivas e correlação de Spearman para identificar fatores significativos, que foram então incluídos como variáveis em um modelo de regressão logística.

**Resultados.:**

Sessenta e oito por cento dos pacientes com malária concluíram o seguimento. A adesão foi superior no centro de saúde maior e mais estabelecido de Léogâne (67%) do que no de Cité Soleil (33%). Os perfis socioeconômicos dos pacientes nos dois centros de saúde divergiram em relação à escolaridade, diversidade religiosa, tamanho da família e porcentagem de indivíduos casados. As análises de regressão logística brutas identificaram a localização do centro de saúde (OR = 0,179 [IC 95% 0,064; 0,504]) e o número de residentes no domicílio (OR = 1,374 [IC 95% 1,056; 1,787]) como fatores associados à adesão. O modelo ajustado identificou apenas a localização do centro de saúde (OR = 0,226 [IC 95% 0,056; 0,918]) como fator significativamente associado à conformidade.

**Conclusão.:**

Embora o número de residentes no domicílio dos pacientes possa ser importante de acordo com a análise de regressão logística bruta, na análise ajustada a localização do centro de saúde onde os pacientes receberam tratamento foi identificada como o único fator importante associado à adesão ao seguimento em pacientes com malária durante o tratamento no Haiti. Essas informações podem ser úteis para melhorar os desfechos do tratamento e contribuir para o monitoramento da resistência aos antimaláricos no Haiti.

Haiti, one of two countries in the Caribbean where endemic malaria still persists (1), continues to use chloroquine (CQ) as the first-line medicine for malaria treatment (2) because widespread CQ resistance has not been reported (3, 4). With increasing concern about the emergence of antimalarial resistance in Haiti, periodic monitoring of treatment outcomes in malaria patients is needed to ensure continued efficacy of CQ for malaria treatment (5).

The identification of antimalarial resistance in patients is important for population-level malaria control (6). The World Health Organization recommends that patients undergoing antimalarial treatment be followed up to monitor completion of treatment regimen and to confirm cure or treatment failure (7). From a population-level perspective, patients not complying with health center staff’s request to complete their treatment regimens can provide a subtherapeutic environment that would facilitate the development of drug resistance in malaria parasites (8, 9). Additionally, patients not complying with physicians’ advice to complete follow-up visits hinders the tracking of emerging antimalarial resistance (10).

The factors associated with treatment follow-up include financial resources (11), side effects of treatment (12), and alleviation of symptoms and persistence of symptoms (13). In addition, physician-specific behaviors and practices such as communication with patients, organizational practices, and their availability outside the clinical setting can impact patient treatment follow-up (11, 14). Although several studies have looked into noncompliance to antimalarial treatment (15), unanswered questions remain about factors that impact follow-up. To begin to investigate this question in Haiti, this study was conducted with confirmed malaria patients who were being treated and followed up in a CQ efficacy study at two health centers. The objective of this study was to identify factors affecting compliance with follow-up during treatment in confirmed malaria patients at two health centers in Haiti.

## MATERIALS AND METHODS

### Study type and location

This was a prospective observational study involving confirmed malaria patients being followed up during treatment with CQ. The study was conducted at two health centers in Haiti. The first was a small non-governmental operated clinic in the neighborhood of Terre Noire, near Cite Soleil, an urban slum community with poor public health infrastructure but close to the Port-au-Prince international airport. The second was a larger health center partially funded by the Ministry of Health of Haiti and located in the coastal town of Leogane, 20 miles west of Port-au-Prince. The Leogane area consists of semiurban/rural communities with a local economy primarily consisting of subsistence farming, fishing, and informal trade. The two health centers represent the most common types of heath care facilities found in Haiti and which have administration styles that could influence participant follow-up.

### Study population and collection of demographic data

The study population was malaria patients enrolled in a CQ efficacy study that was done at the two health centers described above between 2013 and 2015. More information about the CQ efficacy study can be found here (16). Before the study began, the clinic workers at both locations were provided training on the study follow-up protocol and other compliance parameters and were supported through continuous monitoring by the investigators. The demographic data were collected in the questionnaires completed during patient enrollment (16). Additional data were collected after the end of the treatment where patients were followed up to their homes and interviewed. The data collected included age, gender, marital status, household size, education, religion/religious affiliation, and time to nearest health center. The global positioning system (GPS) coordinates of patients’ residences were also collected and used to calculate physical distance (km) to the study clinics. Distance (km) was calculated by inputting GPS coordinates into Google Maps directions application (17).

### Follow-up schedule related to the study

Malaria patients who met enrollment criteria and agreed to participate in the study were asked to comply with a follow-up schedule to monitor symptoms and the decline of parasitemia, as described previously (16). The follow-up schedule required participants to return for follow-up visits on the first, second, and third day, and thereafter every seven days until the end of the study on day 42. At the time of consenting, the patients were also required to fill out a questionnaire that collected demographic and socioeconomic data. During return follow-up visits, the patients presented their study cards to the registry at the health center records department before being seen by the physician and having follow-up tests. To ensure that the patients were compliant to the treatment, the two health centers provided financial support for transportation. In addition, meals were paid for or money was provided to cover one meal, usually lunch, for the patient and guardian, if applicable. In addition, motorcycles were purchased for the health centers and used by community health workers to visit patients at their home and to ferry patients to the health centers, if needed.

### Analysis of patient follow-up (compliance data)

The follow-up (compliance) analysis was conducted on patients with socioeconomic and demographic records and at least day 1 record of treatment. The procedures used to retain patients and data management practices at the health centers were also examined. Two sources were used to obtain the patient follow-up data. The first source was the physicians who examined the registry records and chronicled the patient’s history of return visits. The second source was health center staff (nurses and community health workers) who interviewed patients in person during return visits, by telephone, and after the end of the treatment. The data were categorized into two patient compliance groups: compliant (completed follow-up) and non-compliant (incomplete follow-up) groups. The compliant patients were those who never missed a return visit, while the non-compliant patients (incomplete follow-up) comprised those patients who missed one or more visits, including those that could be contacted by telephone to confirm that they did not complete follow-up and those who could not be contacted and were lost to follow-up.

### Statistical analysis

For the analysis, socioeconomic and demographic data were grouped into continuous and categorical variables. Age, household size, and distance to clinic were grouped under continuous variables, whereas gender, marital status, education, and time to nearest health center were treated as categorical variables in the analysis. Descriptive statistics were used to summarize the socioeconomic and demographic data. Normality of the data was assessed using the Shapiro–Wilk test. Because the data were not normally distributed, Spearman’s rank correlation coefficient was used to evaluate the association of all the variables with compliance. The variables that were significantly correlated based on Spearman’s rank test (*p* < 0.05 and correlation coefficient > 0.2) were used as predictors in a logistic regression model with age and gender as covariates. Odds ratios and their 95% confidence intervals were used to assess association between socioeconomic factors and compliance data. The coefficient of determination (R^2^) was used to assess the overall fit of the logistic regression model. All statistical analyses were done using SAS 9.2 (SAS Institute, Inc., Cary, NC).

### Ethics statement

This study was approved by the Ministry of Health of Haiti National Bioethics Committee, the University of Florida Institutional Review Board (UF-IRB), and the Office of Research Protections, United States Army Medical Research and Material Command (USAMRMC). Written informed consent was obtained from all participants. All the patient records were handled according to the standards required of the UF-IRB and ensured that all patient information was kept anonymously and securely locked and only available to the medical doctors providing care to the patients.

## RESULTS

### Participant follow-up

Overall, 68% (55/81) of the patients completed the follow-up visits and were thus compliant, while 32% (26/81) did not. Of those who did not complete follow-up, 15 (58%) were patients that could be contacted by telephone to confirm that they did not complete follow-up and 11 (42%) were lost to follow-up. The compliance rate was higher in Leogane health center (67%) than in Cite Soleil health center (33%).

### Demographic characteristics

Tables 1 and 2 show the demographic characteristics of the patients in each study site. More males (58%) than females (42%) participated in the study. The mean age of the patients in the study was 33.43 years (range 10–61 years), but the bulk of the patients were between the ages of 18 and 25. More than 44% of participants were single, had at least secondary school education, and identified themselves as Christians (catholic, protestant, or undefined). The average household size was 5.93 ± 2.38 persons and 35.81% of participants reported living 30 min or less away from their nearest clinic. At Leogane, on average, participants lived a distance of 5.72 ± 3.88 km from the nearest health center. Tables 3 and 4 show the demographic data summarized by patients who completed and those who did not complete follow-up. Analysis by Spearman’s rank correlation test showed that study site and household size were significantly associated with patients who completed follow-up (r_s_ = –0.38, *p* < 0.001; and r_s_ = –0.37, *p* < 0.001, respectively). Other significant correlations but not with patient follow-up included travel time to health center, which was correlated with education (r_s_ = –0.28, *p* = 0.03), study site with household size and marital status (r_s_ = –0.27, *p* = 0.04; r_s_ = –0.28, *p* = 0.03, respectively), and marital status with age and education (r_s_ = 0.525, *p* < 0.001; r_s_ = –0.40, *p* = 0.002, respectively).

### Regression analysis

The results of the logistic regression analysis are presented in Table 5. Although the crude logistic regression model showed that health center (study site) and household size were associated with follow-up compliance (site OR = 0.179 [95% CI 0.064, 0.504] and household size OR = 1.374 [95% CI 1.056, 1.787]), the adjusted model identified health center (study site) as the only significant factor associated with completing follow-up (OR = 0.226 [95% CI 0.056, 0.918]) (Table 5). Analysis by study site showed that household size was not significantly associated with follow-up compliance.

**TABLE 1.** Descriptive statistics of socioeconomic and demographic categorical variables by study site

Variable		Cite Soleil clinic		Leogane clinic		Total	
		n	%	n	%	N	%
All patients		37	45.68	44	54.32	81	100.00
Sex	Female	16	43.24	18	40.91	34	41.98
	Male	21	56.76	26	59.09	47	58.02
	Total	37	100.00	44	100.00	81	100.00
Age	<18	2	5.41	7	15.91	9	11.11
	18–25	15	40.54	10	22.73	25	30.86
	26–35	5	13.51	12	27.27	17	20.99
	36–45	9	24.32	4	9.09	13	16.05
	46–55	3	8.11	3	6.82	6	7.41
	56–65	3	8.11	5	11.36	8	9.88
	>65	0	0.00	3	6.82	3	3.70
	Total	37	100.00	44	100.00	81	100.00
Marital status	Single	17	45.95	19	43.18	36	44.44
	Married	12	32.43	2	4.55	14	17.28
	Other	7	18.92	2	4.55	9	11.11
	Missing data	1	2.70	21	47.73	22	27.16
	Total	37	100.00	44	100.00	81	100.00
Education	Primary school	0	0.00	2	4.55	2	2.47
	<Secondary school	16	43.24	3	6.82	19	23.46
	Secondary school	13	35.14	16	36.36	29	35.80
	University	7	18.92	2	4.55	9	11.11
	Missing data	1	2.70	21	47.73	22	27.16
	Total	37	100.00	44	100.00	81	100.00
Religion	Protestant	31	83.78	12	27.27	43	53.09
	Catholic	4	10.81	7	15.91	11	13.58
	Voodoo	0	0.00	2	4.55	2	2.47
	Jehovah’s Witness	0	0.00	1	2.27	1	1.23
	No religion	1	2.70	0	0.00	1	1.23
	Missing data	1	2.70	22	50.00	23	28.40
	Total	37	100.00	44	100.00	81	100.00
Time to nearest clinic	<15 min	7	18.92	3	6.82	10	12.35
	15–30 min	11	29.73	8	18.18	19	23.46
	31–60 min	12	32.43	9	20.45	21	25.93
	Over 1 hour	5	13.51	0	0.00	5	6.17
	Missing data	2	5.41	24	54.55	26	32.10
	Total	37	100.00	44	100.00	81	100.00

***Source:*** Prepared by the authors from the results of this study.

**TABLE 2.** Mean and standard deviation for continuous variables by study site

Variable	Cite Soleil clinic		Leogane clinic		Total	
	n	Mean ± SD	n	Mean ± SD	N	Mean ± SD
Age (y)	37	32.14 ± 12.85	44	34.52 ± 17.99	81	33.43 ± 15.80
Household size	36	6.5 ± 2.59	23	5.04 ± 1.72	59	5.93 ± 2.38
Distance to clinic (km)	--	--	24	5.72 ± 3.88	24	5.72 ± 3.88

***Source***: Prepared by the authors from the results of this study.

**TABLE 3.** Descriptive statistics for socioeconomic variables of patients who were compliant with follow-up (n = 55) and those who were not (n = 26)

**Variable**		**Compliant**		**Non-compliant**		**Total**	
		**n**	**%**	**n**	**%**	**N**	**%**
Site	Cite Soleil clinic	18	32.73	19	73.08	37	45.68
	Leogane clinic	37	67.27	7	26.92	44	54.32
Sex	Female	21	38.18	13	50.00	34	41.98
	Male	34	61.82	13	50.00	47	58.02
Age	<18	6	10.91	3	11.54	9	11.11
	18–25	17	30.91	8	30.77	25	30.86
	26–35	12	21.82	5	19.23	17	20.99
	36–45	7	12.73	6	23.08	13	16.05
	46–55	3	5.45	3	11.54	6	7.41
	56–65	8	14.55	0	0.00	8	9.88
	>65	2	3.64	1	3.85	3	3.70
Marital status	Single	23	41.82	13	50.00	36	44.44
	Married	7	12.73	7	26.92	14	17.28
	Other	7	12.73	2	7.69	9	11.11
	Missing data	18	32.73	4	15.38	22	27.16
Education	Primary school	2	3.64	0	0.00	2	2.47
	<Secondary school	8	14.55	11	42.31	19	23.46
	Secondary school	21	38.18	8	30.77	29	35.80
	University	6	10.91	3	11.54	9	11.11
	Missing data	18	32.73	4	15.38	22	27.16
Religion	Christian, other	25	45.45	18	69.23	43	53.09
	Christian, Catholic	9	16.36	2	7.69	11	13.58
	Voodoo	2	3.64	0	0.00	2	2.47
	Jehovah’s Witness	0	0.00	1	3.85	1	1.23
	No religion	1	1.82	0	0.00	1	1.23
	Missing data	18	32.73	5	19.23	23	28.40
Time to clinic	<15 min	6	10.91	4	15.38	10	12.35
	15–30 min	13	23.64	6	23.08	19	23.46
	31–60 min	12	21.82	9	34.62	21	25.93
	Over 1 hour	2	3.64	3	11.54	5	6.17
	Missing data	22	40.00	4	15.38	26	32.10

***Source***: Prepared by the authors from the results of this study.

**TABLE 4.** Mean and standard deviation for continuous variables by follow-up group

Variable	Compliant		Non-compliant		Total	
	n	Mean ± SD	n	Mean ± SD	N	Mean ± SD
Age (y)	55	34.36 ± 16.85	26	31.33 ± 14.23	81	33.43 ± 15.80
Household size	37	5.32 ± 2.27	22	6.95 ± 2.26	59	5.93 ± 2.38
Distance to clinic (km)	20	6.09 ± 3.89	4	3.86 ± 3.73	24	5.72 ± 3.88

***Source***: Prepared by the authors from the results of this study.

**TABLE 5.** Logistic regression models for patients not completing follow-up

Predictor	Crude		Adjusted	
	OR (95% CI)	R^2^	OR (95% CI)	R^2^
Age (continuous)	0.988 (0.958, 1.019)	0.0076	0.982 (0.934, 1.033)	0.2173
Gender (ref. female)	0.618 (0.241, 1.584)	0.0123	0.309 (0.087, 1.098)	
Site (ref. Leogane)	0.179 (0.064, 0.504)	0.1361	0.226 (0.056, 0.918)	
Household size (continuous)	1.374 (1.056, 1.787)	0.1080	1.336 (0.999, 1.787)	

***Source***: Prepared by the authors from the results of this study.

## DISCUSSION

This study was conducted to identify factors affecting follow-up compliance in malaria patients undergoing treatment in Haiti. The study identified the study site (health center) as the most important factor associated with follow-up compliance. The overall compliance rate of 68% may suggest that most patients were committed to the study and that malaria was taken seriously by the patients. The observed differences in compliance rates between the two study sites led us to examine site-specific demographic factors as well as patient retention and management processes at the study clinics.

There were interesting and important demographic aspects about the groups of patients in this study. Participants were mostly young, between the ages of 18 and 25 years, which may reflect the younger age profiles in the general population of Haiti. In addition, most of the study participants were single, as would be expected in a younger population. The fact that most of the participants had secondary school education at a higher rate than the general population (18) is interesting and may suggest that the more educated individuals were more concerned with their health and were more likely to seek treatment. However, the participants were not explicitly asked on this aspect of the question. Most participants were Christians and this likely reflects that most Haitians are religious, with Christianity being the most widespread religion in Haiti.

The demographic profiles were different at each health center study site, but they were not of significance for follow-up compliance. Compared with the Cite Soleil health center, patients recruited at the health center in Leogane, where compliance rates were high, had more education, were more religiously diverse, had smaller household sizes, and the majority were unmarried. However, except for household size, all the other demographic and socioeconomic factors were not associated with follow-up compliance in crude regression analysis. Some studies have observed that highly educated participants are more likely to complete antimalarial treatment (15). However, in our study, it appeared that the educational level did not affect follow-up completion. Several factors that could have diminished the impact of education include the fact that medications and other testing services were provided free of charge, as well as the population being in an under-resourced community that was in need of health care services. Additionally, the high unemployment rates could have been a driving force that encouraged participants to come back for follow-up, sometimes in the hope that they would be able to build connections with the study team, which might help them find employment opportunities.

The management and retention practices of the health center study managers could also have played a role in observed follow-up compliance rates, although this was not analyzed in the regression model. These practices included reminders for participants about their upcoming appointments via phone calls, cash assistance for transportation and taxis (“tap-tap”), and home visits by community health agents on motorcycle to pick up the participants. The increased attention and effort aimed at retaining the patients resulted in higher follow-up compliance rates. For example, in the health center in Leogane, participants were reimbursed the money they spent on transportation and meals after their final follow-up visit in week 6. However, in the health center in Cite Soleil, reimbursement was given out after each follow-up visit. It is not clear if this practice may have caused the follow-up compliance rate differential between the two study sites, but it is possible that the practice of distributing the reimbursement at the end of the study was a better incentive for patients to return for every visit until the very end. Other retention practices may also indirectly influence the associations, or lack thereof, observed between the socioeconomic variables and follow-up compliance. For instance, the lack of association between distance to clinic and compliance in the health center in Leogane may be related to the transportation assistance made available. If patients received free rides to the health center site, then the potential obstacle of distance to clinic may be eliminated. As for the management and documentation practices, both clinics were provided laptops for record-keeping; however, it is not clear if computer competency in the clinics was an issue, as we did not specifically study it. It is very likely that the medical doctors (with an M.D. degree) would have been competent in the use of a computer for data entry. It is also possible that the nurses or other record management team members may have been undertrained or could not complete the records as needed, which presents a potential area for improvement in future similar studies.

There were several limitations in this study, including the small sample of participants with complete data. There were 40 patients who voluntarily withdrew (requested to be removed) from the efficacy study (16) and their data were not used or included in this study. In addition, there were some incomplete surveys due to the participants not showing up for follow-up visits. Also, the GPS coordinates that would have been useful in determining the distance participants traveled to clinics were missing in many records, particularly for the health center in Cite Soleil, and therefore we were unable to use the Cite Soleil distance data. Another limitation was with how the records were managed: the health care center in Leogane had organized data in an Excel spreadsheet but the Cite Soleil site did not keep complete spreadsheets. In addition, there were several missing patient study records, incorrectly completed forms, and duplicate participant identification numbers at the Cite Soleil site, which might have led to loss of patient follow-up. All this points to organizational difficulties at the site. These problems could have been the primary cause of the lower follow-up rates. The observational nature of the study is another limitation because we can only infer a relationship between variables and not causation. Finally, there are concerns related to the transfer of survey information from the participant to the investigator. Because the information was passed through multiple parties (participant to health center staff to the study’s principal investigator), it is possible that some of the open-ended survey data might not have been interpreted correctly.

Despite the limitations, the results of the study highlight important sociodemographic characteristics of the Haitian participants and the potential role that study sites play in achieving high compliance in patient follow-up visits. The study lays the groundwork for future studies to explore site-specific and patient-specific characteristics that can improve monitoring of antimalarial resistance and malaria patient treatment in Haiti and elsewhere.

Based on the findings of this study, we would recommend that the Ministry of Health and other public health funders should consider providing support for the development of good practices for documentation of patient records in health centers across the country. These records can be used in similar studies monitoring treatment compliance and even to track patients who might present with drug resistant parasites. Such records might be key for identifying and even preventing the spread of drug resistant malaria.

## Disclaimer.

Authors hold sole responsibility for the views expressed in the manuscript, which may not necessarily reflect the opinion or policy of the *RPSP/PAJPH* and/or those of the Pan American Health Organization, or the views of the Uniformed Services University of Health Sciences or the Department of Defense. Mention of trade names, commercial products, or organizations does not imply endorsement by the U.S. Government.
